# AOTMiT reimbursement recommendations compared to other HTA agencies

**DOI:** 10.1007/s10198-023-01655-x

**Published:** 2024-01-23

**Authors:** Aneta Mela, Dorota Lis, Elżbieta Rdzanek, Janusz Jaroszyński, Marzena Furtak-Niczyporuk, Bartłomiej Drop, Tomasz Blicharski, Maciej Niewada

**Affiliations:** 1 Department of Experimental and Clinical Pharmacology, Centre for Preclinical Research and Technology (CePT), Medical University of Warsaw, Banacha 1B, 02-097 Warsaw, Poland; 2 Department of Administrative Procedure, Faculty of Law and Administration, Maria Curie-Skłodowska University of Lublin, Marii Curie-Skłodowskiej 5, 20-031 Lublin, Poland; 3 Department of Public Health, Medical University of Lublin, Chodźki 1, 20-093 Lublin, Poland; 4 Department of Orthopeadics and Rehabilitation, Medical University of Lublin, K. Jaczewskiego 8, 20-090 Lublin, Poland; 5 HealthQuest Sp z o.o. Sp. K, 01-625 Warsaw, Poland; 6 Department of Information Technology and Medical Statistics, Faculty of Health Sciences, Medical University of Lublin, 20-093 Lublin, Poland

**Keywords:** Reimbursement recommendations, AHTAPol, HTA agencies, AOTMiT

## Abstract

Our objective was to compare AOTMiT (Polish: Agencja Oceny Technologii Medycznych i Taryfikacji) recommendations to other HTA (Health Technology Assessment) agencies for newly registered drugs and new registration indications issued by the European Medicines Agency between 2014 and 2019. The study aims to assess the consistency and justifications of AOTMiT recommendations compared to that of other HTA agencies in 11 countries. A total of 2496 reimbursement recommendations published by 12 HTA agencies for 464 medicinal products and 525 indications were analyzed. Our analysis confirmed that the Polish AOTMiT agency seems to bear the closest resemblance to the corresponding HTA agencies from Canada (CADTH) and New Zealand (PHARMAC), when it comes to the outcome of HTA recommendations (positive or negative). Poland had a general scheme for justifying recommendations, similar to that of Ireland—four aspects (i.e., clinical efficacy, safety profile, cost-effectiveness, and impact on the payer’s budget) are important for Poland when formulating the final decision. Compared to other countries, Poland shows a noticeably different pattern of justifying reimbursement recommendations, as revealed primarily in terms of budget impact and somewhat less so for cost-effectiveness rationales.

## Introduction

In the course of the debate on potentially harmonizing and centralizing HTA (Health technology assessment) to avoid duplication of assessment, the substantial research on HTA transferability in Europe indicates that it may be possible to strive for a unified evaluation of clinical efficacy at this stage. In contrast, economic assessments should remain within the competence of the national agencies. Since 2004, many organizations dealing with HTA in Europe have been interconnected through a network of European organizations working in the field of HTA (EUnetHTA). Key activities undertaken by EUnetHTA for the HTA network include the development of principles for European technology assessment in clinical performance. These actions are designed to reduce the burden at the national level and to make it easier for HTA bodies in the member states to conduct additional analyses and make decisions specific to a given healthcare system [1].

Bearing in mind the different management models of HTA agencies, the differing nature of the issued recommendations or stances (positive, negative, conditional), placing a drug on either the (positive) reimbursement list or the (negative) list of products that are not reimbursed, with the consequently unequal global access to innovative pharmacotherapy implies, the following research questions can be posed: Are the differences in the recommendations issued by HTA agencies for the same drugs used for the same indications significant or rather negligible? What are the reasons for the differences in recommendations issued by HTA agencies in particular therapeutic areas—are they determined by different interpretations and evaluations of clinical data or economic reasons? Answers to these questions should indeed be sought by carrying out a comprehensive substantive analysis of the recommendations of HTA agencies across Europe and the world regarding individual therapeutic areas. Following the example of other developed countries, Poland introduced the health technology assessment (HTA) system in 2005 to the drug reimbursement process by establishing the Agency for Health Technology Assessment [2]. In 2009, the Agency for Health Technology Assessment (Polish: Agencja Oceny Technologii Medycznych) received statutory authorization and subsequently changed its name to the Agency for Health Technology Assessment and Tariff System (AOTMiT) (Polish: Agencja Oceny Technologii Medycznych i Taryfikacji). AOTMiT is an independent organizational unit that collects data, performs analyses, and issues independent recommendations on the legitimacy of public funding of medicines, medical devices, foodstuffs for special nutritional purposes, as well as healthcare services [3]. The collected analytical data and recommendations of AOTMiT support the Minister of Health in making reimbursement decisions [4]. According to the guidelines of the president of the Agency, any drug manufacturer, in addition to analyzing the decision problem, must present four types of analyses: a clinical efficacy analysis, an economic analysis, a health system impact analysis and a rationalization analysis. The first of these analyses answers the question regarding what additional clinical benefits concerning the currently used standard in a given indication will the introduction of a new therapy bring. The second analysis assesses cost-effectiveness—it provides an answer to the question regarding the profitability of treatment. The Polish legislator precisely specifies the situation in which the reimbursement of a new therapy is cost-effective compared to the alternative form of treatment. This happens when the cost-effectiveness ratio does not exceed the willingness to pay threshold set at the level of three times the GDP per capita. The third of the analyses concerns assessing the impact of introducing a new medical technology on the payer's budget. It makes it possible to determine what additional expenses the payer will have to incur as a consequence of introducing a new therapy into clinical practice. The last one, a rationalization analysis that is specific only to Poland, should be developed if the budget impact analysis indicates extra spending by the public payer and aims to identify reallocation of the current budget to accommodate additional anticipated costs [5].

The main goal of this work is a systematic quantitative analysis of AOTMiT recommendations compared to other HTA agencies in particular therapeutic areas, taking into account the division into newly registered drugs and new registration indications issued by the European Medicines Agency (EMA) between 2014 and 2019. The study aims to assess the consistency and justifications of AOTMiT recommendations compared to other HTA agencies.

## Materials and methods

A structured database was designed to extract the data from the reimbursement recommendations published by individual HTA agencies for medicinal products (new molecules or indications) registered by the EMA from 2014 to 2019.

We included HTA agencies which were explicitly quoted in recommendations issued by AOTMiT does not publish a formal list of reference agencies, thus we decided to identify them based on practice and published recommendations. The following were covered: the National Institute for Health and Care Excellence (NICE) in England; the Scottish Medicines Consortium (SMC) in Scotland; the All Wales Medicines Strategy Group (AWMSG) in Wales; the National Centre for Pharmacoeconomics (NCPE) in Ireland; Haute Autorité de Santé (HAS) in France; the National Health Care Institute (NHCI) in the Netherlands; the Federal Joint Committee (Gemeinsamer Bundesausschuss) (G-BA) in Germany; the Norwegian Medicines Agency (NoMA) and Norwegian Institute of Public Health (FHI) in Norway; the Canadian Agency for Drugs & Technologies in Health (CADTH) in Canada; the Pharmaceutical Benefits Scheme (PBS) in Australia; the Pharmaceutical Management Agency (PHARMAC) in New Zealand.

The search for reimbursement recommendations on the websites of agencies was carried out from 2018 to 2021 by two HTA specialists. Since the reimbursement recommendations in many countries are published with a considerable delay after the registration date, indications authorized by the EMA between 2014 and 2019 were deliberately selected to increase the likelihood of these recommendations being available. Consequently, to capture all the relevant recommendations, the related websites were searched and the database updated several times. The last update was carried out from October 2021 to January 2022.

The database was designed after a pilot analysis of the selected recommendations for all of the countries covered. Finally, the following data were collected: therapeutic area indication, orphan designation, recommendation date, recommendation type, comparator and intervention attributes, e.g., clinical efficacy, safety profile, cost-effectiveness, and impact on the payer's budget. The attributes were coded and interpreted as shown in Table 6 in the Appendix. It should be stressed that we report separately as to whether the specific information was available and how it was interpreted and used to support the final recommendation. Consequently, to assess the consistency of HTA agencies’ reimbursement recommendations, we adopted two main analyses:

1. A quotation analysis—whether the HTA agency refers to a given aspect (i.e., clinical benefit, safety profile, cost-effectiveness, budgetary impact) in its recommendation.
2. Appraisal analysis—whether an individual aspect was explicitly judged by the HTA agency to be favorable or not, and how it impacted its recommendation.

The adopted approach resulted from the way we interpreted the recommendations. The presence or absence of particular information in the recommendation was easy to extract and report. On the other hand, unambiguous interpretation of whether particular data were perceived as favorable/attractive or not, and explicitly used to justify the recommendation, was not always feasible. Consequently, the appraisal analysis was based on a smaller data set, which solely consists of distinct and clear recommendations, not only reporting data but interpreting it and explicitly using it to justify the final decision. Inadequate data resulted in the cross-countries and therapeutic areas analyses being limited to a quotation analysis.

Additionally, HTA agencies were compared among themselves (each country with every other country for the same indications for the compared pairs) regarding the type of reimbursement recommendation issued. Furthermore, the Polish HTA agency was compared with other agencies in terms of justifying recommendations, i.e., referring to specific assessment aspects and covering clinical efficacy, safety profile, cost-effectiveness, and budget impact assessment.

Due to skewed distributions, prevalence and bias adjusted kappa (PABAK [6, 7]) was used instead of Cohen's kappa for conformity assessment between pairs of countries regarding their reimbursement recommendations, as well as the reference and value of the aspects for clinical benefit, safety, cost-effectiveness, and budget impact.

Descriptive statistics were provided; odds ratios were calculated along with estimates of 95% confidence intervals using the Wald method. Regarding quotation analyses, the odds ratio was derived by dividing the odds of a positive recommendation, among cases characterized by a specific aspect, by the odds of a positive recommendation among those without said aspect. An analogous approach was used in the appraisal analysis—comparing the odds of a positive recommendation, among those who appreciated an individual aspect, to the odds of a positive recommendation among those who underestimated it.

Analyses were conducted individually for each of the four aspects. In cases involving incomplete information (i.e., when at least one subgroup had no observations), the sole aim was to obtain odds ratio estimates. To achieve this, the observation count within each of the four aspects was augmented by one.

The analyses were performed with R 4.1.3 in the RStudio integrated development environment (build 443).

## Results

**Fig. 1:**
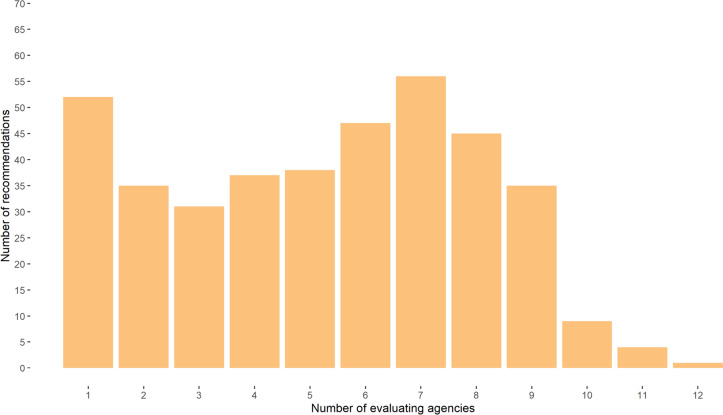
Number of recommendations evaluated in 2014–2019 in relation to the number of agencies

**Fig. 2:**
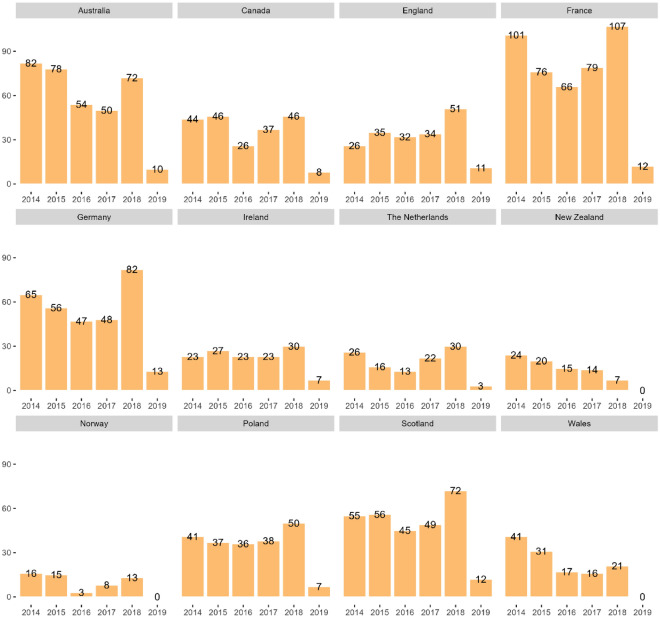
Number of evaluated recommendations in 2014–2019 by agency per country

A total of 2496 reimbursement recommendations published by 12 HTA agencies for 464 medicinal products and 525 indications were analyzed (Table 1, Figs. 1, 2 in the Appendix).

**Table 1:** Number of drugs and indications authorized between 2014 and 2019 and covered in the analysis

Year	Number of entities	Number of indications	Number of recommendations
2014	73	82	544
2015	94	99	493
2016	85	105	377
2017	97	101	418
2018	106	124	581
2019	9	14	83
Total	**464**	**525**	**2496**

All of the countries, except Wales and Ireland, published more positive reimbursement recommendations than negative ones. For Poland, Germany, and Australia, the positive recommendations rate was only marginally higher than the negative one (Appendix, Table 7). The therapeutic areas in which agencies much more frequently published positive recommendations included diabetology, cardiology, pulmonology, neurology, ophthalmology, psychiatry, dermatology, gynecology, infectious diseases, and immunology. For oncology, rare diseases, gastroenterology, urology, and nephrology, positive recommendations were made slightly less frequently (Appendix, Table 8)). It should be noted, however, that all the analyzed countries, except for Australia, Ireland and Wales issued positive reimbursement recommendations for oncological medicinal products and drugs used in rare diseases more frequently (Appendix, Table 9).

### Conformity assessment of reimbursement recommendations issued by different agencies

A moderate agreement for the type of reimbursement recommendations made in the same indications for the compared pairs of HTA agencies was observed. The agencies in Scotland and Canada (showing alignment with 9 agencies) were the most aligned with other agencies, followed by France and the Netherlands (aligned with 7 agencies). To a lesser extent, alignment was also observed for England, Australia (with 6 agencies), Poland, and New Zealand (with 5 agencies). However, countries such as Norway, Germany, and Wales had differing assessments of the same drugs, showing alignment with individual countries only. The agency in Ireland exhibited the highest level of divergence—no alignment was observed with any of the analyzed agencies (Table 2).

**Table 2:**
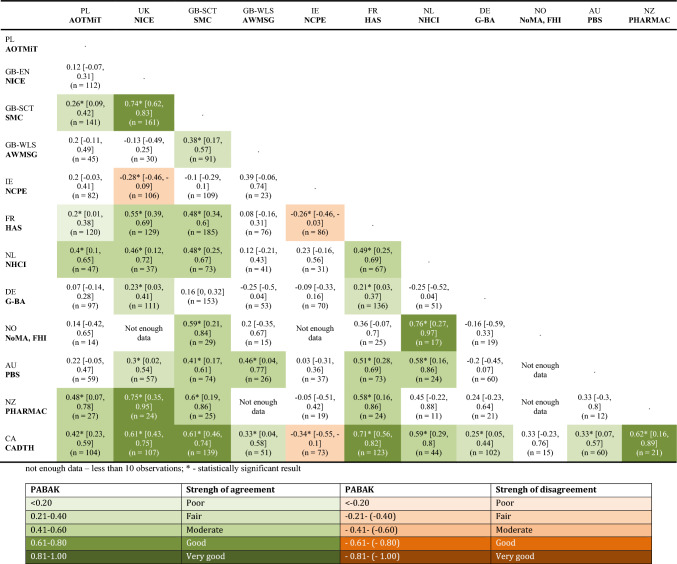
Analysis of the consistency of HTA agencies in terms of the type of reimbursement recommendations—comparison of each country with every other country within common indications for the compared pairs

Agency for Health Technology Assessment and Tariff System (AOTMiT) in Poland, National Institute for Health and Care Excellence (NICE) in England, Scottish Medicines Consortium (SMC) in Scotland, All Wales Medicines Strategy Group (AWMSG) in Wales, National Centre Pharmacoeconomics (NCPE) in Ireland, Haute Autorité de Santé (HAS) in France, National Health Care Institute (NHCI) in the Netherlands, The Federal Joint Committee (Gemeinsamer Bundesausschuss) (G-BA) in Germany, The Norwegian Medicines Agency (NoMA) and Norwegian Institute of Public Health (FHI) in Norway, Canadian Agency for Drugs & Technologies in Health (CADTH) in Canada, Pharmaceutical Benefits Scheme (PBS) in Australia, The Pharmaceutical Management Agency (PHARMAC) in New Zealand

Table 2 presents PABAK coefficients, indicating the degrees of agreement between pairs of countries. Each cell illustrates the agreement estimate between the country in the corresponding row and the country in the corresponding column.

### Conformity assessment between AOTMIT and other HTA agencies—findings from quotation and appraisal analyses

Among the comparable pairs, focusing on the same indications, when assessing clinical data for reimbursement recommendations, Poland demonstrated concurrence with half of the examined nations. These aligned countries encompass England, Scotland, Ireland, France, Germany, and Canada (Table 3).

**Table 3:**
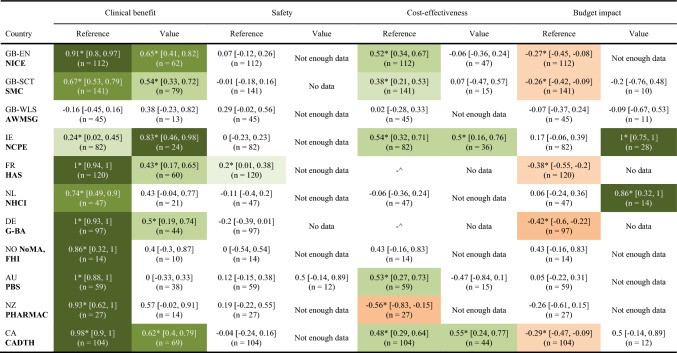
Analysis of consistency in the justification of recommendations—comparison of the Polish agency with other HTA agencies

No data value = no data reference

*Statistically significant result

^Insufficient references

**Fig. 3:**
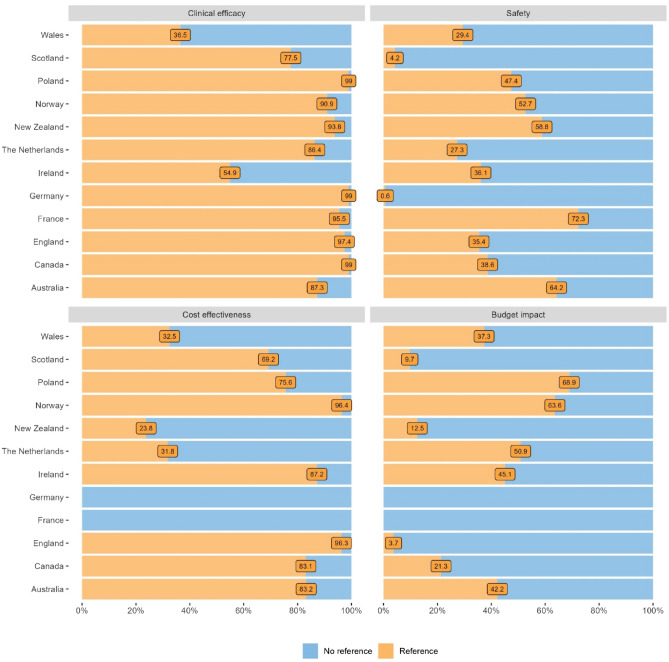
Percentage of quotations (references) by agency per country

**Fig. 4:**
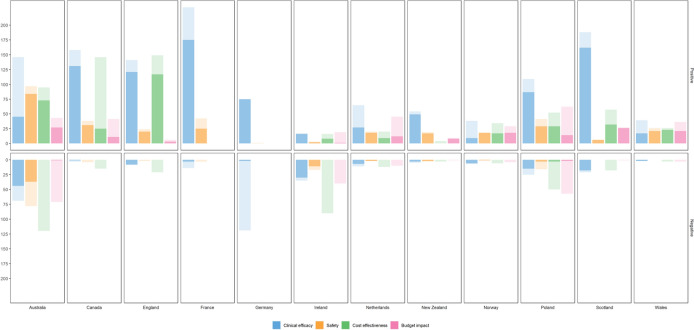
Percentage of favorable and unfavorable appraisals (ratings) within positive and negative recommendations. Favorable—intense color; unfavorable—bright color

Clinical efficacy rationales were cited significantly more often in positive and less often in negative recommendations in Scotland and Wales (Table 4). Clinical benefits were used as justification for recommendations primarily in Germany, and to a lesser extent in France and Poland (Table 5). On the contrary, in Norway and Australia, favorable recommendations were probable, despite uncertainties surrounding clinical benefits. Conversely, unfavorable recommendations were issued even when clinical benefits were acknowledged, implying the influence of other criteria in the decision-making process (Table 5, Figs. 3 and 4 in the Appendix).

**Table 4:** Quotation analysis of four aspects by agencies

Agency	Recommendation	Clinical benefit			Safety			Cost-effectiveness			Budget impact		
		No	Yes	Odds ratio	No	Yes	Odds ratio	No	Yes	Odds ratio	No	Yes	Odds ratio
PL **AOTMiT** (*n* = 209)	Negative	1	82	1.52 [0.09, 24.71]	50	33	1.67 [0.95, 2.92]	16	67	0.62 [0.32, 1.21]	18	65	0.47 [0.25, 0.88]^b^
	Positive	1	125		60	66		35	91		47	79	
GB-EN **NICE** (*n* = 189)	Negative	0	21	1.24 [0.14, 10.81]^1^	17	4	2.55 [0.82, 7.92]	0	21	0.92 [0.11, 7.71]^1^	20	1	0.74 [0.08, 6.47]
	Positive	5	163		105	63		7	161		162	6	
GB-SCT **SMC** (*n* = 289)	Negative	46	26	18.44 [9.41, 36.14]^a^	72	0	4.61 [0.59, 35.84]^1^	45	27	6.55 [3.67, 11.71]^a^	71	1	10.09 [1.35, 75.64]^a^
	Positive	19	198		205	12		44	173		190	27	
GB-WLS **AWMSG** (*n* = 126)	Negative	71	4	82.83 [24.02, 285.68]^a^	72	3	48 [13.17, 174.96]^a^	69	6	25.16 [9.05, 69.95]^a^	71	4	95.41 [27.1, 335.87]^a^
	Positive	9	42		17	34		16	35		8	43	
IE **NCPE** (*n* = 133)	Negative	46	52	1.33 [0.61, 2.91]	61	37	0.76 [0.33, 1.72]	6	92	0.14 [0.05, 0.42]^b^	58	40	1.93 [0.89, 4.22]
	Positive	14	21		24	11		11	24		15	20	
FR **HAS** (*n* = 441)	Negative	2	49	0.84 [0.19, 3.75]	16	35	1.22 [0.65, 2.3]	51	0	0.13 [0.01, 2.16]^1^	51	0	0.13 [0.01, 2.16]^1^
	Positive	18	372		106	284		390	0		390	0	
NL **NHCI** (*n* = 110)	Negative	2	20	0.58 [0.12, 2.77]	17	5	1.35 [0.45, 4.05]	7	15	0.14 [0.05, 0.38]^b^	12	10	1.31 [0.51, 3.36]
	Positive	13	75		63	25		68	20		42	46	
DE **G-BA** (*n* = 311)	Negative	3	121	6.16 [0.68, 55.8]^1^	123	1	0.66 [0.04, 10.67]	124	0	0.66 [0.04, 10.73]^1^	124	0	0.66 [0.04, 10.73]^1^
	Positive	0	187		186	1		187	0		187	0	
NO **NoMA, FHI** (*n* = 55)	Negative	1	7	1.54 [0.15, 15.82]	3	5	0.63 [0.13, 2.92]	0	8	1.7 [0.16, 18.28]^1^	4	4	1.94 [0.43, 8.78]
	Positive	4	43		23	24		2	45		16	31	
AU **PBS** (*n* = 346)	Negative	18	147	0.73 [0.38, 1.39]	56	109	0.85 [0.55, 1.33]	19	146	0.47 [0.26, 0.86]^b^	79	86	0.46 [0.29, 0.7]^b^
	Positive	26	155		68	113		39	142		121	60	
NZ **PHARMAC** (*n* = 80)	Negative	0	11	0.9 [0.1, 8.19]^1^	2	9	0.27 [0.05, 1.35]	6	5	0.31 [0.08, 1.15]	10	1	1.5 [0.17, 13.16]
	Positive	5	64		31	38		55	14		60	9	
CA **CADTH** (*n* = 207)	Negative	0	32	1.76 [0.18, 17.42]^1^	24	8	2.1 [0.89, 4.93]	17	15	9.89 [4.23, 23.09]^a^	32	0	11.25 [1.5, 84.64]^a1^
	Positive	2	173		103	72		18	157		131	44	

Agency for Health Technology Assessment and Tariff System (AOTMiT) in Poland, National Institute for Health and Care Excellence (NICE) in England, Scottish Medicines Consortium (SMC) in Scotland, All Wales Medicines Strategy Group (AWMSG) in Wales, National Centre Pharmacoeconomics (NCPE) in Ireland, Haute Autorité de Santé (HAS) in France, National Health Care Institute (NHCI) in the Netherlands, The Federal Joint Committee (Gemeinsamer Bundesausschuss) (G-BA) in Germany, The Norwegian Medicines Agency (NoMA) and Norwegian Institute of Public Health (FHI) in Norway, Canadian Agency for Drugs & Technologies in Health (CADTH) in Canada, Pharmaceutical Benefits Scheme (PBS) in Australia, The Pharmaceutical Management Agency (PHARMAC) in New Zealand

^1^odds-ratio estimations included one additional observation for each cell

^a^statistically significant result—positive recommendations referred to the aspect more often than negative recommendations

^b^statistically significant result—positive recommendations referred to the aspect less often compared to negative recommendations

*N* number of evaluated recommendations in 2014–2019 by agency per country

**Table 5:** Appraisal analysis of four aspects by agencies

Agency	Recommendation	Clinical benefit			Safety			Cost-effectiveness			Budget impact		
		–	+	Odds ratio	–	+	Odds ratio	–	+	Odds ratio	–	+	Odds ratio
PL **AOTMiT**	Negative	10	15	2.64 [1.04, 6.66]^a^	13	3	10.47 [2.52, 43.51]^a^	47	3	19.75 [5.44, 71.69]^a^	55	2	8.02 [1.73, 37.09]^a^
	Positive	22	87		12	29		23	29		48	14	
GB-ENG **NICE**	Negative	1	8	0.76 [0.09, 6.38]	2	0	12.6 [1.07, 148.13]^a1^	21	0	78.67 [10.22, 605.5]^a1^	0	0	1 [0.05, 22.18]^1^
	Positive	20	121		4	20		32	117		3	3	
GB-SCT **SMC**	Negative	3	18	1.04 [0.29, 3.77]	0	0	7 [0.22, 226]^1^	18	0	24.12 [3.03, 192.17]^a1^	1	0	27 [1.65, 442.83]^a1^
	Positive	26	162		0	6		25	32		1	26	
GB-WLS **AWMSG**	Negative	0	2	0.26 [0.02, 2.72]^1^	0	0	3.67 [0.2, 67.65]^1^	3	0	24 [2.11, 273.59]^a1^	3	0	5.5 [0.56, 53.99]^1^
	Positive	22	17		5	21		3	23		15	21	
IE **NCPE**	Negative	5	30	2.67 [0.29, 24.83]	6	11	1.09 [0.08, 14.66]	90	0	91 [10.32, 802.23]^a1^	40	0	4.32 [0.37, 50.58]^1^
	Positive	1	16		1	2		8	8		18	1	
FR **HAS**	Negative	11	3	11.67 [3.14, 43.33]^a^	3	0	5.78 [0.6, 56.05]^1^	0	0	–	0	0	–
	Positive	55	175		17	25		0	0		0	0	
NL **NHCI**	Negative	4	7	0.41 [0.11, 1.53]	0	2	2.11 [0.16, 27.58]^1^	12	0	10.83 [1.2, 97.8]^a1^	10	0	4.21 [0.49, 35.91]^1^
	Positive	38	27		2	18		11	9		33	12	
DE **G-BA**	Negative	117	2	2989.33 [305.32, 29,268.37]^a1^	0	0	0.5 [0.01, 19.56]^1^	0	0	–	0	0	–
	Positive	0	75		1	0		0	0		0	0	
NO **NoMA, FHI**	Negative	1	6	0.05 [0.01, 0.49]^b^	0	1	9.5 [0.41, 217.61]^1^	6	0	7 [0.78, 62.85]^1^	4	0	7.92 [0.82, 76.28]^1^
	Positive	29	9		0	18		17	17		11	18	
AU **PBS**	Negative	25	44	0.25 [0.14, 0.46]^b^	41	37	7.16 [3.44, 14.92]^a^	120	0	389.3 [51.49, 2943.24]^a1^	70	1	118.12 [14.93, 934.72]^a^
	Positive	101	45		13	84		22	73		16	27	
NZ **PHARMAC**	Negative	2	3	6.53 [0.87, 48.86]	1	2	4.25 [0.26, 70.75]	3	0	0.8 [0.04, 17.2]^1^	1	0	9 [0.52, 155.24]^1^
	Positive	5	49		2	17		4	0		1	8	
CA **CADTH**	Negative	2	1	9.7 [0.85, 110.88]	4	0	20 [2.04, 196.03]^a1^	15	0	3.41 [0.43, 26.87]^1^	0	0	0.39 [0.02, 6.7]^1^
	Positive	27	131		7	31		121	25		30	11	

Agency for Health Technology Assessment and Tariff System (AOTMiT) in Poland, National Institute for Health and Care Excellence (NICE) in England, Scottish Medicines Consortium (SMC) in Scotland, All Wales Medicines Strategy Group (AWMSG) in Wales, National Centre Pharmacoeconomics (NCPE) in Ireland, Haute Autorité de Santé (HAS) in France, National Health Care Institute (NHCI) in the Netherlands, The Federal Joint Committee (Gemeinsamer Bundesausschuss) (G-BA) in Germany, The Norwegian Medicines Agency (NoMA) and Norwegian Institute of Public Health (FHI) in Norway, Canadian Agency for Drugs & Technologies in Health (CADTH) in Canada, Pharmaceutical Benefits Scheme (PBS) in Australia, The Pharmaceutical Management Agency (PHARMAC) in New Zealand

^1^Odds ratio estimations included one additional observation for each cell

^a^Statistically significant result—positive recommendations were more likely to show within the aspect benefit than negative recommendations

^b^Statistically significant result—positive recommendations were less likely to show a benefit within the aspect compared to negative recommendations

In terms of analyzing AOTMIT alignment with other countries regarding referencing and assessing the safety profile, it was not possible to draw appropriate conclusions due to insufficient data in this aspect (Table 3).The safety profile was significantly more frequently quoted in positive recommendations than negative ones in Wales alone (Table 4). Safety was used significantly more to consistently justify positive and negative recommendations by the agencies in Poland, England, Canada, and to a slightly lesser extent by Australia (Table 5, Figs. 3 and 4 in the Appendix).

Regarding cost-effectiveness, Poland was moderately aligned with England, Scotland, Ireland, Australia, and Canada. However, in terms of assessing cost-effectiveness across the five countries for which data were obtained, alignment was observed only for Ireland and Canada (Table 3).

In Ireland, the Netherlands and Australia, cost-effectiveness was mentioned more often in negative recommendations than positive ones. Both recommendations were consistently justified with this aspect (i.e., the positive recommendations referred to attractive cost-effectiveness and the negative ones to the contrary). It can be argued that cost-effectiveness in these countries seems to support negative recommendations more commonly than positive ones. The opposite was noted in Wales and Scotland: cost-effectiveness was more likely revealed and used to justify positive recommendations. In Poland and England, cost-effectiveness was likely to be pointed out regardless of recommendation outcomes and used consistently to justify both positive and negative recommendations (Tables 4, 5, Figs. 3, 4 in the Appendix).

Poland exhibits a noticeably different pattern of justifying reimbursement recommendations, which becomes evident through the analysis of the budget impact as a specific decision criterion. Regarding the assessment of budget impact, the obtained results should be interpreted with a high degree of caution due to the small sample sizes involved. Nonetheless, in two cases—concerning Ireland and the Netherlands—Poland demonstrated high alignment (Table 3).

Analogous to cost-effectiveness, the budget impact was used more to explain negative recommendations in Poland and Australia and (once again) the positive ones in Scotland. In Wales and Canada, the budget impact was more likely to be quoted in positive recommendations, but not used to justify the final recommendations (Tables 4, 5, Figs. 3, 4 in the Appendix).

### Quotation analysis by therapeutic areas

**Fig. 5:**
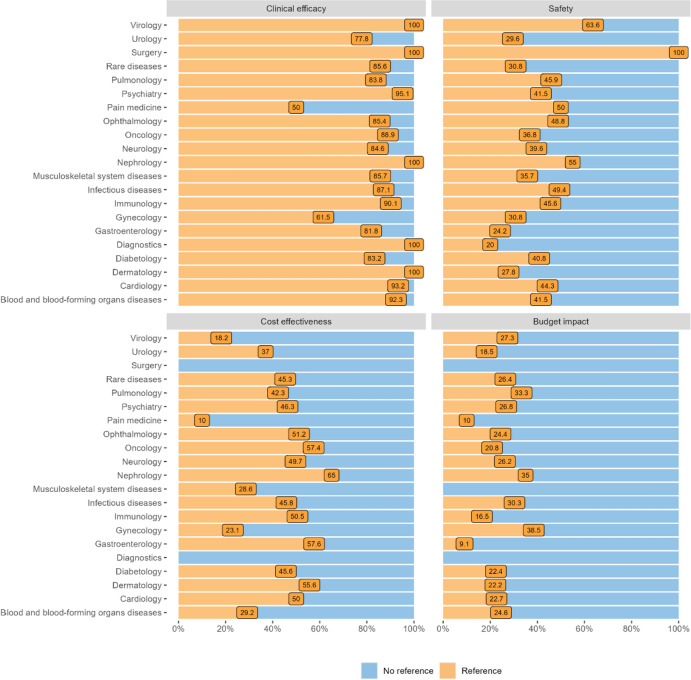
Percentage of quotations (references) by therapeutic area

Out of the considered criteria, clinical efficacy was quoted most often in positive than negative recommendations across different therapeutic areas (especially for drugs used in gynecology, gastroenterology, urology and pain medicine). Safety was only quoted in a few areas, mainly pain medicine. Economic aspects, both cost-effectiveness and affordability, were likewise reported more often for positive than negative recommendations in diabetology and infectious diseases. In contrast, for oncology and blood disorders, economic criteria were more commonly invoked for negative recommendations than positive ones. Additionally, drugs for neurology and particularly nephrology, were questioned in the context of their budget impact. Economic criteria, as compared to clinical ones, were cited more often in negative recommendations, especially for therapeutic areas with many new highly priced innovative drugs or for potential use by a large number of patients, and thus resulting in prohibitive budgetary expenditure (Appendix, Table 10), Fig. 5 in the Appendix).

## Discussion

The present paper addresses the similarities, differences and justifications of reimbursement recommendations published by 12 HTA agencies for new molecules and indications authorized in 2014–2019, with a total of about 2,500 reimbursement recommendations across various therapeutic areas issued by agencies in Poland, England, Scotland, Wales, Ireland, France, Germany, the Netherlands, Norway, Canada, Australia, and New Zealand.

Similar topics have also been covered in other publications, all of which have been concerned with both a smaller number of countries and recommendations included in the comparison, as well as a narrower or different range of aspects analyzed.

Currently, according to the HTA guidelines, the common elements of HTA assessment in each country include unmet medical needs, degree of innovation, clinical efficacy, and safety profile although the last two are the key ones. Cost-effectiveness and impact on the payer’s budget are also important for most agencies—only the French and German agencies do not formally consider these criteria [8–12].

In this study, we observed that while individual countries often refer to clinical efficacy, safety profile, cost-effectiveness, and budget impact, these references do not consistently lead to the precise assignment of a particular value, and subsequently they do not always dictate the type of reimbursement recommendation ultimately provided.

As indicated in the publication by Niewada et al. [13], who analyzed recommendations published before October 7, 2011, the process of making reimbursement recommendations by the AOTMiT is multi-criteria, and its outcomes are not easily predictable. The study demonstrated that clinical efficacy and safety significantly influenced the final recommendations. In contrast, the cost-effectiveness of the evaluated therapy and its impact on the payer's budget were less frequently utilized to justify the ultimate decisions taken. However, the present study reveals that economic aspects, namely cost-effectiveness and budget impact assessment, are gaining increasing importance for the Polish HTA agency. Consequently, similar to clinical efficacy, they substantially contribute to the final recommendation.

Non-specific results were reported for agencies in Canada, Norway, and New Zealand. The statistically significant positive recommendations from the Canadian Agency were primarily influenced by the safety profile. The Norwegian agency, on the other hand, published negative recommendations despite a favorable assessment of clinical efficacy, suggesting that they were guided more by economic criteria, albeit rather inconsistently (as showed by statistically insignificant OR). Likewise, for New Zealand, where statistical significance was not achieved for all aspects, the agency nevertheless referred to clinical efficacy most often in its rationale in almost all recommendations. The latter two countries’ findings may result from the small number of observations (reimbursement recommendations that were published in the time horizon analyzed), which affects interpretation (Appendix, Tables 11, 12).

Agreement in valuing clinical efficacy is evident between HTA agencies in France and Germany. Similar concordance was observed by Schaefer et al., who evaluated recommendations for 102 drugs, published by three HTA agencies, i.e., NICE (England), G-BA (Germany), and HAS (France). Higher concordance was confirmed for G-BA/HAS (67% concordance in total, 72% concordance for oncology drugs, and 59% concordance for non-oncology drugs) than for G-BA/NICE and HAS/NICE (54% concordance in total, 57% concordance for oncology drugs and 50% concordance for non-oncology drugs) [14].

For the other agencies analyzed, significant discrepancies are apparent in the referencing and valuing of aspects in the justification of reimbursement recommendations. These differences are due to several reasons:

1. First, the criteria for selecting a drug for which an HTA is being carried out vary from agency to agency.

In Poland all newly registered drugs and new indications are evaluated. In contrast, in the UK, for example, all drugs are potentially reimbursed after marketing approval; of the drugs approved each year, only a small proportion are evaluated based on a number of selection criteria, including the likely high impact on NHS resources [15].

2) Second, limited evidence is available, and long-term data in particular are lacking, which is a cause of uncertainty and inconsistency.

Individual HTA agencies may respond in different ways to evidence gaps, and this varies between countries. They may focus on a particular subpopulation of patients or a narrow indication for which the evidence is more reliable and may draw on indirect comparisons and expert opinions to fulfill evidence requirements as well as by deferring a decision until additional evidence is received, by rejecting the application, by influencing the manufacturer to lower its prices, or by introducing risk-sharing agreements.

In Poland, the recommendations of the President of the AOTMiT are not binding. Indeed, decisions made by the Minister of Health could be influenced by changes that occur at later stages of the process, such as the drug prices negotiations. Nonetheless, the results of an AOTMiT assessment in Poland can have a significant impact on the course of price negotiations. However, in countries such as Wales, Ireland, the Netherlands, and Canada, where agencies perform an advisory function, positive reimbursement recommendations do not always translate into the reimbursement of a given technology in the final coverage, and their impact on the outcome of price negotiations is unclear. HTA agencies have individual discretion regarding the methodology used for the indirect comparison of treatments. Only the G-BA emphasizes a need for direct evidence and a requirement for convincing arguments to justify not having head-to-head data [11, 14, 16].

3) Third, divergent acknowledgments of the quality and robustness of clinical trial evidence lead to variations in HTA outcomes [12, 13].

This was confirmed by Zhou et al. who evaluated reimbursement recommendations for the same 15 medicinal products, published by agencies in England, Scotland, Canada, and Australia in 2017–2018. Poor agreement was observed between England, Scotland, and Canada (− 0.41 < kappa score < 0.192). Canada placed more emphasis on open-label trials and cost–utility analysis, while Australia took less account of the results of economic models. Australia had a much greater preference for direct RCTs and indirect comparisons, while placebo comparisons were listed as acceptable evidence by England and Scotland [17].

Moreover, Nicod and Kanavos (2010) pointed out that there is considerable variability between countries in the HTA recommendations published between 2007 and 2009 for the same 25 drug products by the agency in England, Wales, Ireland, Scotland, France, Sweden, Australia, and Canada. Forty-six percent of the drug pairs surveyed received divergent recommendations. The level of inter-agency agreement was poor to moderate. The link between HTA recommendations made by each HTA body for therapeutic areas such as oncology, neurology, and rare disease treatment differed from the general pattern observed in the sample as a whole. It also turned out that divergent interpretations of the same evidence cause differences in HTA results [14, 15].

- 4) Fourth, a key criterion that apparently differentiates the various agencies on HTA is cost-effectiveness [18].

In Canada, Australia, and New Zealand there is no officially set value below which a drug is considered cost-effective [19]. Poland is an example of a country where the regulation defines exactly when reimbursement of a new therapy is cost-effective. In addition, the NICE (England) approach is most in favor of entering into risk-sharing agreements and negotiating prices as early as during the HTA review to reach the cost-effectiveness threshold adopted by the agency [20].

Vreman et al. analyzed recommendations published by England, France, Germany, the Netherlands, and Scotland for 27 drugs registered between 2006 and 2016. The inclusion of cost-effectiveness evaluations over the years led to a significant increase in the percentage of negative recommendations in England (from 4 to 50%), and Scotland (from 21 to 71%). In contrast, the subsequent introduction of price negotiations led to a significant reduction in the percentage of negative recommendations in England (from 50 to 14%), France (from 31 to 3%), and Germany (from 34 to 0%). No correlation was observed in the Netherlands, probably due to the sample size [21].

- 5) Fifth, an approach to evaluating recommendations for innovative oncology products and orphan drugs.

In contrast to Poland, higher cost-effectiveness thresholds for cancer and orphan drugs are accepted in certain countries, such as England, Scotland, or Canada [22]. In Germany, on the other hand, documentation of additional clinical benefits is not required for orphan drugs. This results in a higher chance of a negative reimbursement recommendation in countries that have more stringent criteria for evaluating these therapeutic areas [23].

Stawowczyk et al. assessed the status of reimbursement recommendations for orphan drugs in various countries, including Belgium, England, France, Germany, Poland, Scotland, and Spain and found the highest percentage of negative recommendations in Poland (49%) [24].

Despite a generally consistent approach to conducting health technology assessments across countries, this study highlights the significant level of divergence in reimbursement recommendations made for medicinal products in the 12 countries analyzed. HTA methods may be influenced by different priorities across conditions, different preferences based on individual conditions and therapeutic areas, levels of evidence hierarchy, perceptions of value, tools used to address uncertainty, and the ability and willingness to implement risk-sharing agreements.

Established in 2005, the EUnetHTA initiative, the mission of which is to foster cooperation among European institutions involved in HTA assessment, will likely minimize discrepancies in methods used and clinical data interpretation as economic findings most likely remain country specific. An approach in which a single clinical assessment shared by many countries, is prepared will significantly reduce the burden on individual countries, which should have a positive impact on reducing the time to reimbursement. On the other hand, the development of analyses that can be easily adapted to local conditions will allow these analyses to be tailored to the differences found in the various reimbursement systems [1].

While many healthcare systems utilize HTA to support reimbursement decisions, its implementation is not uniform. Inconsistencies can arise due to the nature of the final decision-making process, which can either be stepwise, incremental, involving multiple stakeholders, an integrated process, or a decision made independently by the responsible body (usually the Ministry of Health, as is the case in Poland), based on recommendations or statements from other auxiliary bodies.

Undoubtedly, these various approaches can lead to differences in the time required to make decisions and in patients’ access to medications. However, it should be noted that the time elapsed from registration to patient access is lengthy, not only due to delays caused by institutions involved in the reimbursement process but also as a result of reimbursement budget constraints allocated by individual countries. Additionally, it can result from the strategy adopted by pharmaceutical companies, which may not necessarily initiate the process simultaneously in every country and only sometimes immediately after marketing authorization.

## Limitations

The present analysis is not without limitations. (1) The first is the inclusion of only 12 countries in the analysis, but this is the result of the approach taken, in which the subject of the analysis was to compare the Polish HTA agency with the countries referred to by the Polish HTA agency. The agency in Sweden (SBU, TLV) was not included in the analysis. This was due to the fact that the Polish agency rarely refers to the Swedish recommendations, as well as the SBU focusing more on evaluating available scientific evidence and developing its own systematic reviews, while the TLV issues reimbursement decisions for individual medicinal products with limited access to detailed information on the rationale behind these recommendations. (2) An important limitation of the analysis is that the recommendations found within the 12 countries did not always refer to the same drug. Figure 1 indicates that data were obtained from all agencies in the study only for one indication. (3) Another limitation is the small number of recommendations found for Norway and New Zealand, which causes interpretive difficulties for the results within those countries. (4) Other interpretive difficulties arise from the fact that failure to address aspect in the rationale for recommendations does not imply that they were not important to the HTA agency in question. (5) In addition, due to the small number of observations within each therapeutic area undertaken by the agencies analyzed, it was impossible to assess in detail the convergence or divergence of countries in this regard.

## Conclusions

1. The outcome of HTA recommendations (positive or negative) in Poland bears greater resemblance to that of Canada or New Zealand than to numerous other European countries.
2. Poland had the most similar general pattern of justifying recommendations to Ireland—four aspects are important for Poland when formulating the final decision.
3. However, compared to other countries, Poland shows a noticeably different pattern of justifying reimbursement recommendations as revealed mainly in terms of budget impact and less so for cost-effectiveness rationales.

## Figures and Tables

**Table 11:**
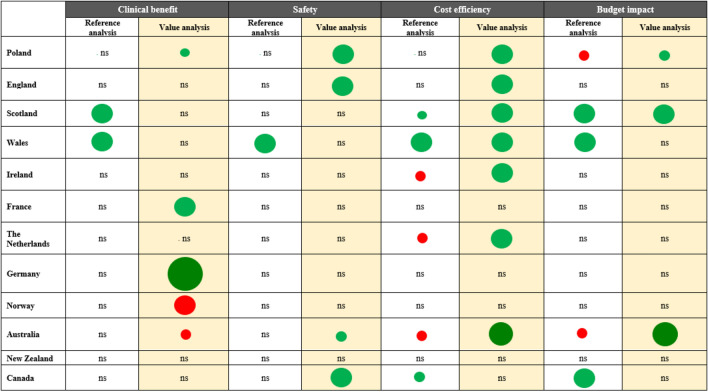
Summary of the results of the quotation (reference) and appraisal (value) analysis by agency

**Table 12:**
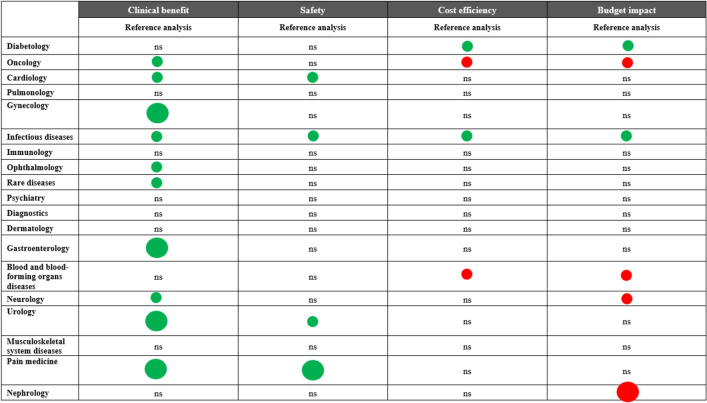
Summary of the results of the quotation (reference) analysis by therapeutic area

## Results: overall

(Figs. 1,2 and Tables 6, 7, 8, 9).

**Table 6:** Information collected within the database

Aspect	Coding	Favorable	Unfavorable
Recommendation type	0—negative 1—positive 9—no recommendation	–	–
Recommended indication/population	1—on label 2—restricted label	1—on label	2—restricted label
Comparator	1—well selected − 1—poorly selected 9—no information	1—well selected	− 1—poorly selected
Clinical efficacy	1—an advantage was shown − 1—no advantage was shown 0—cannot be stated unequivocally 9—no information	1—an advantage was shown	− 1—no advantage was shown
Safety profile	1—better safety profile − 1—comparable safety profile − 2—worse safety profile 0—cannot be determined 9—no information	1—better safety profile -1—comparable safety profile	-2—worse safety profile
Cost-effectiveness	1—an advantage was shown (lower cost in CMA and cost-effectiveness in CEA, CUA) − 1—no advantage was shown (uncertainty as to the analysis parameters) − 2)—ineffective in terms of cost 2—uncertainty 0—cannot be determined 9—no information	1—an advantage was shown (lower cost in CMA and cost-effectiveness in CEA, CUA)	-1—no advantage was shown (uncertainty as to the analysis parameters) − 2)—ineffective in terms of cost 2—uncertainty
Impact on the payer's budget	1—savings − 1—an additional burden 0—neutral 2—uncertainty − 2—cannot be determined 9—no information	1—savings 0—neutral	− 1—an additional burden 2—uncertainty

**Table 7:** Number of evaluated positive and negative recommendations in 2014–2019 by agency per country

Agency	Positive (%)	Negative (%)	Total
Poland	126 (60.3)	83 (39.7)	209
England	168 (88.9)	21 (11.1)	189
Scotland	217 (75.1)	72 (24.9)	289
Wales	51 (40.5)	75 (59.5)	126
Ireland	35 (26.3)	98 (73.7)	133
France	390 (88.4)	51 (11.6)	441
The Netherlands	88 (80.0)	22 (20.0)	110
Germany	187 (60.1)	124 (39.9)	311
Norway	47 (85.5)	8 (14.5)	55
Australia	181 (52.3)	165 (47.7)	346
New Zealand	69 (86.2)	11 (13.8)	80
Canada	175 (84.5)	32 (15.5)	207

**Table 8:** Number of evaluated positive and negative recommendations in 2014–2019 by therapeutic area

Therapeutic area	Positive (%)	Negative (%)	Total
Blood and blood-forming organs’ diseases	43 (66.2)	22 (33.8)	65
Cardiology	65 (73.9)	23 (26.1)	88
Dermatology	14 (77.8)	4 (22.2)	18
Diabetology	89 (71.2)	36 (28.8)	125
Diagnostics	3 (60)	2 (40)	5
Gastroenterology	20 (60.6)	13 (39.4)	33
Gynecology	9 (69.2)	4 (30.8)	13
Immunology	135 (74.2)	47 (25.8)	182
Infectious diseases	217 (80.1)	54 (19.9)	271
Musculoskeletal system diseases	9 (64.3)	5 (35.7)	14
Nephrology	11 (55)	9 (45)	20
Neurology	101 (67.8)	48 (32.2)	149
Oncology	711 (66.4)	359 (33.6)	1,070
Ophthalmology	30 (73.2)	11 (26.8)	41
Pain medicine	6 (60)	4 (40)	10
Psychiatry	30 (73.2)	11 (26.8)	41
Pulmonology	89 (80.2)	22 (19.8)	111
Rare diseases	123 (61.2)	78 (38.8)	201
Surgery	1 (100)	0 (0)	1
Urology	17 (63)	10 (37)	27
Virology	11 (100)	0 (0)	11

**Table 9:** Number of evaluated positive and negative recommendations in 2014–2019 by oncology and rare diseases and per country

	Australia (%)	Canada (%)	France (%)	Germany (%)	Ireland (%)	Netherlands (%)	New Zealand (%)	Norway (%)	Poland (%)	Scotland (%)	England (%)	Wales (%)
Oncology												
Negative	82 (52.9)	18 (19.8)	19 (11.9)	53 (34.9)	57 (79.2)	7 (25)	8 (17.4)	1 (50)	40 (39.6)	32 (25.6)	16 (15)	26 (81.2)
Positive	73 (47.1)	73 (80.2)	140 (88.1)	99 (65.1)	15 (20.8)	21 (75)	38 (82.6)	1 (50)	61 (60.4)	93 (74.4)	91 (85)	6 (18.8)
Rare diseases												
Negative	18 (78.3)	5 (26.3)	3 (9.1)	5 (19.2)	10 (83.3)	6 (35.3)	0	1 (16.7)	7 (50)	12 (54.5)	2 (15.4)	9 (64.3)
Positive	5 (21.7)	14 (73.7)	30 (90.9)	21 (80.8)	2 (16.7)	11 (64.7)	2 (100)	5 (83.3)	7 (50)	10 (45.5)	11 (84.6)	5 (35.7)

## Results: HTA agency

(Figs. 3, 4, 5, and Table 10).

**Table 10:** Analysis for quotation by therapeutic area

Agency	Recommend	Clinical benefit			Safety			Cost-effectiveness			Budget impact		
		No	Yes	Odds ratio	No	Yes	Odds ratio	No	Yes	Odds ratio	No	Yes	Odds Ratio
Diabetology (*n* = 125)	Neg	8	28	1.67 [0.63, 4.46]	25	11	1.86 [0.81, 4.23]	29	7	5.31 [2.11, 13.4]^a^	33	3	4.3 [1.21, 15.29]^a^
	Pos	13	76		49	40		39	50		64	25	
Oncology (*n* = 1070)	Neg	91	268	8.28 [5.3, 12.94]^a^	241	118	1.3 [0.99, 1.69]	134	225	0.72 [0.55, 0.93]^b^	253	106	0.47 [0.35, 0.64]^b^
	Pos	28	683		435	276		322	389		594	117	
Cardiology (*n* = 88)	Neg	4	19	6.63 [1.13, 39.06]^a^	17	6	2.92 [1.02, 8.35]^a^	11	12	0.89 [0.34, 2.3]	18	5	1.08 [0.34, 3.4]
	Pos	2	63		32	33		33	32		50	15	
Pulmonology (*n* = 111)	Neg	3	19	0.78 [0.2, 2.97]	11	11	0.82 [0.32, 2.08]	9	13	0.43 [0.17, 1.11]	15	7	1.09 [0.4, 2.96]
	Pos	15	74		49	40		55	34		59	30	
Gynecology (*n* = 13)	Neg	4	0	22.5 [1.61, 314.56]*^1^	4	0	4.17 [0.36, 48.44]^1^	4	0	2.86 [0.24, 33.9]^1^	4	0	6 [0.52, 69.75]^1^
	Pos	1	8		5	4		6	3		4	5	
Infectious diseases (*n* = 271)	Neg	18	36	5.88 [2.77, 12.48]^a^	37	17	2.55 [1.35, 4.8]^a^	40	14	2.94 [1.51, 5.71]^a^	45	9	2.53 [1.17, 5.47]^a^
	Pos	17	200		100	117		107	110		144	73	
Immunology (182)	Neg	8	39	2.56 [0.95, 6.95]	30	17	1.69 [0.85, 3.35]	26	21	1.37 [0.7, 2.68]	35	12	0.45 [0.2, 1.02]
	Pos	10	125		69	66		64	71		117	18	
Ophthalmology (*n* = 41)	Neg	4	7	8 [1.21, 52.88]^a^	6	5	1.2 [0.3, 4.8]	5	6	0.83 [0.21, 3.33]	7	4	0.44 [0.1, 2]
	Pos	2	28		15	15		15	15		24	6	
Rare diseases (*n* = 201)	Neg	20	58	4.37 [1.87, 10.2]^a^	56	22	1.23 [0.66, 2.28]	39	39	0.73 [0.41, 1.3]	52	26	0.56 [0.3, 1.06]
	Pos	9	114		83	40		71	52		96	27	
Psychiatry (*n* = 41)	Neg	1	10	2.9 [0.17, 50.82]	7	4	1.34 [0.32, 5.56]	4	7	0.38 [0.09, 1.59]	9	2	1.93 [0.35, 10.77]
	Pos	1	29		17	13		18	12		21	9	
Diagnostics (*n* = 5)	Neg	0	2	1.33 [0.06, 31.12]^1^	1	1	0.25 [0.01, 4.73]^1^	2	0	0.75 [0.03, 17.51]^1^	2	0	0.75 [0.03, 17.51]^1^
	Pos	0	3		3	0		3	0		3	0	
Dermatology (*n* = 18)	Neg	0	4	3 [0.16, 57.36]^1^	2	2	0.27 [0.03, 2.83]	2	2	1.33 [0.14, 12.37]	3	1	0.82 [0.06, 11]
	Pos	0	14		11	3		6	8		11	3	
Gastroenterology (*n* = 33)	Neg	5	8	11.88 [1.19, 118.5]^a^	11	2	2.36 [0.4, 14.04]	3	10	0.25 [0.05, 1.17]	12	1	1.33 [0.11, 16.39]
	Pos	1	19		14	6		11	9		18	2	
Blood and blood-forming organs diseases (*n* = 65)	Neg	1	21	0.46 [0.05, 4.43]	13	9	1.04 [0.37, 2.95]	12	10	0.32 [0.1, 0.97]^b^	13	9	0.28 [0.09, 0.91]^b^
	Pos	4	39		25	18		34	9		36	7	
Neurology (*n* = 149)	Neg	13	35	3.38 [1.36, 8.41]^a^	32	16	1.48 [0.72, 3.04]	23	25	0.87 [0.44, 1.72]	28	20	0.32 [0.15, 0.69]^b^
	Pos	10	91		58	43		52	49		82	19	
Urology (*n* = 27)	Neg	5	5	16 [1.5, 171.2]^a^	10	0	9.9 [1.06, 92.66]^a1^	7	3	1.63 [0.31, 8.61]	9	1	2.77 [0.26, 29.05]
	Pos	1	16		9	8		10	7		13	4	
Musculoskeletal system diseases (*n* = 14)	Neg	1	4	2 [0.1, 41]	3	2	0.75 [0.08, 7.21]	4	1	2 [0.15, 26.73]	5	0	0.6 [0.03, 11.47]^1^
	Pos	1	8		6	3		6	3		9	0	
Pain medicine (*n* = 10)	Neg	4	0	15 [1.03, 218.3]^a1^	4	0	15 [1.03, 218.3]^a1^	4	0	1.67 [0.11, 24.26]^1^	4	0	1.67 [0.11, 24.26]^1^
	Pos	1	5		1	5		5	1		5	1	
Nephrology (*n* = 20)	Neg	0	9	1.2 [0.07, 21.72]^1^	3	6	0.42 [0.07, 2.58]	2	7	0.34 [0.05, 2.46]	3	6	0.05 [0, 0.6]^b^
	Pos	0	11		6	5		5	6		10	1	

^1^Odds ratio estimations included one additional observation for each cell

^a^Statistically significant result—positive recommendations referred to aspect more often than negative recommendations

^b^Statistically significant result—positive recommendations referred to aspect less often compared to negative recommendations

*n* number of evaluated recommendations in 2014–2019 by therapeutic area

## Summary of the results of the quotation (reference) and appraisal (value) analysis by agency and therapeutic area

(Tables 11, 12).

A legend to summarize the results
