# Correction: AOTMiT reimbursement recommendations compared to other HTA agencies

**DOI:** 10.1007/s10198-024-01720-z

**Published:** 2024-10-26

**Authors:** Aneta Mela, Dorota Lis, Elżbieta Rdzanek, Janusz Jaroszyński, Marzena Furtak-Niczyporuk, Bartłomiej Drop, Tomasz Blicharski, Maciej Niewada

**Affiliations:** 1 Department of Experimental and Clinical Pharmacology, Centre for Preclinical Research and Technology (CePT), Medical University of Warsaw, Banacha 1B, Warsaw, 02-097 Poland; 2 HealthQuest Sp z o.o. Sp. K, Warsaw, 01-625 Poland; 3 Department of Administrative Procedure, Faculty of Law and Administration, Maria Curie-Skłodowska University of Lublin, Marii Curie-Skłodowskiej 5, Lublin, 20-031 Poland; 4 Department of Public Health, Medical University of Lublin, Chodźki 1, Lublin, 20-093 Poland; 5 Department of Information Technology and Medical Statistics, Faculty of Health Sciences, Bartłomiej Drop, Medical University of Lublin, Lublin, 20-093 Poland; 6 Department of Orthopeadics and Rehabilitation, Medical University of Lublin, K. Jaczewskiego 8, Lublin, 20-090 Poland

**Correction: The European Journal of Health Economics**

10.1007/s10198-023-01655-x

In the sentence beginning “A total of 2494 reimbursement recommendations…” of the Abstract and Results section in this article, the value “2494” should have read “2496”. In Figs. 2 and 3 of this article, the country name was incorrectly given as “Netherlands” but should have appeared as “The Netherlands”. For completeness and transparency, the old incorrect and the corrected versions are displayed below.

**Incorrect version of Figure 2**

**Correct version of Figure 2**

**Incorrect version of Figure 3**

**Correct version of Figure 3**

**Fig. 2:**
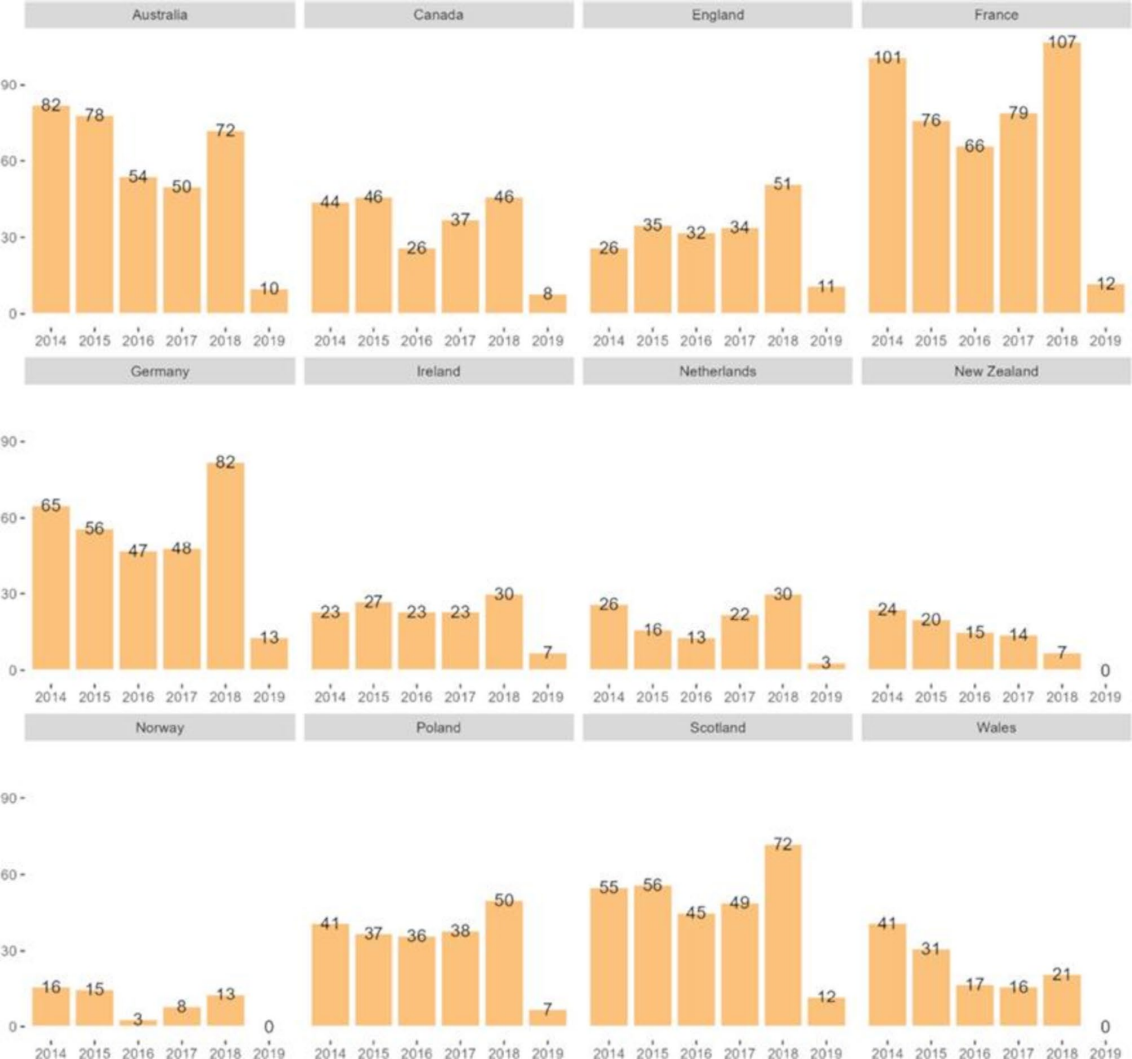
Number of evaluated recommendations in 2014–2019 by agency per country

**Fig. 2:**
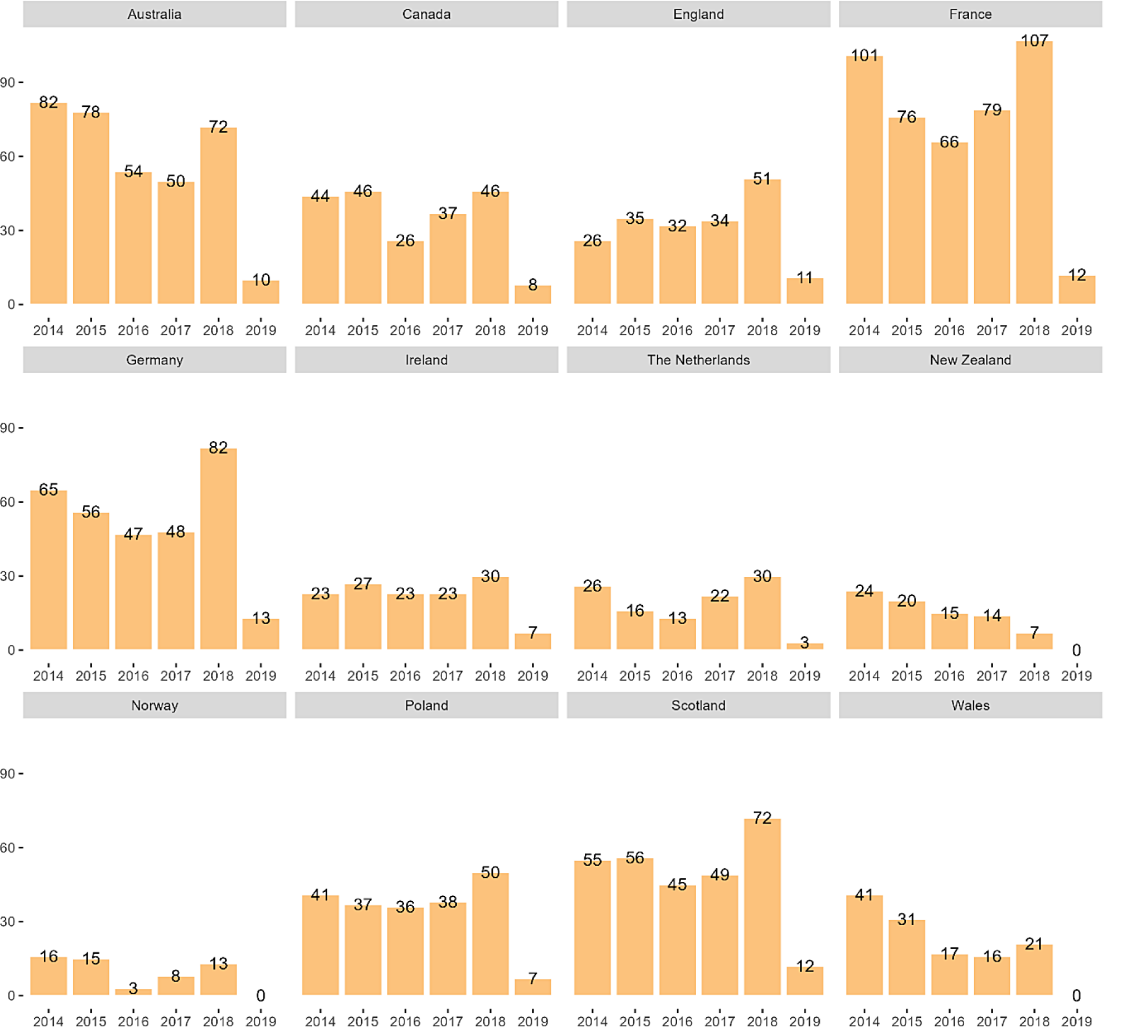
Number of evaluated recommendations in 2014–2019 by agency per country

**Fig. 3:**
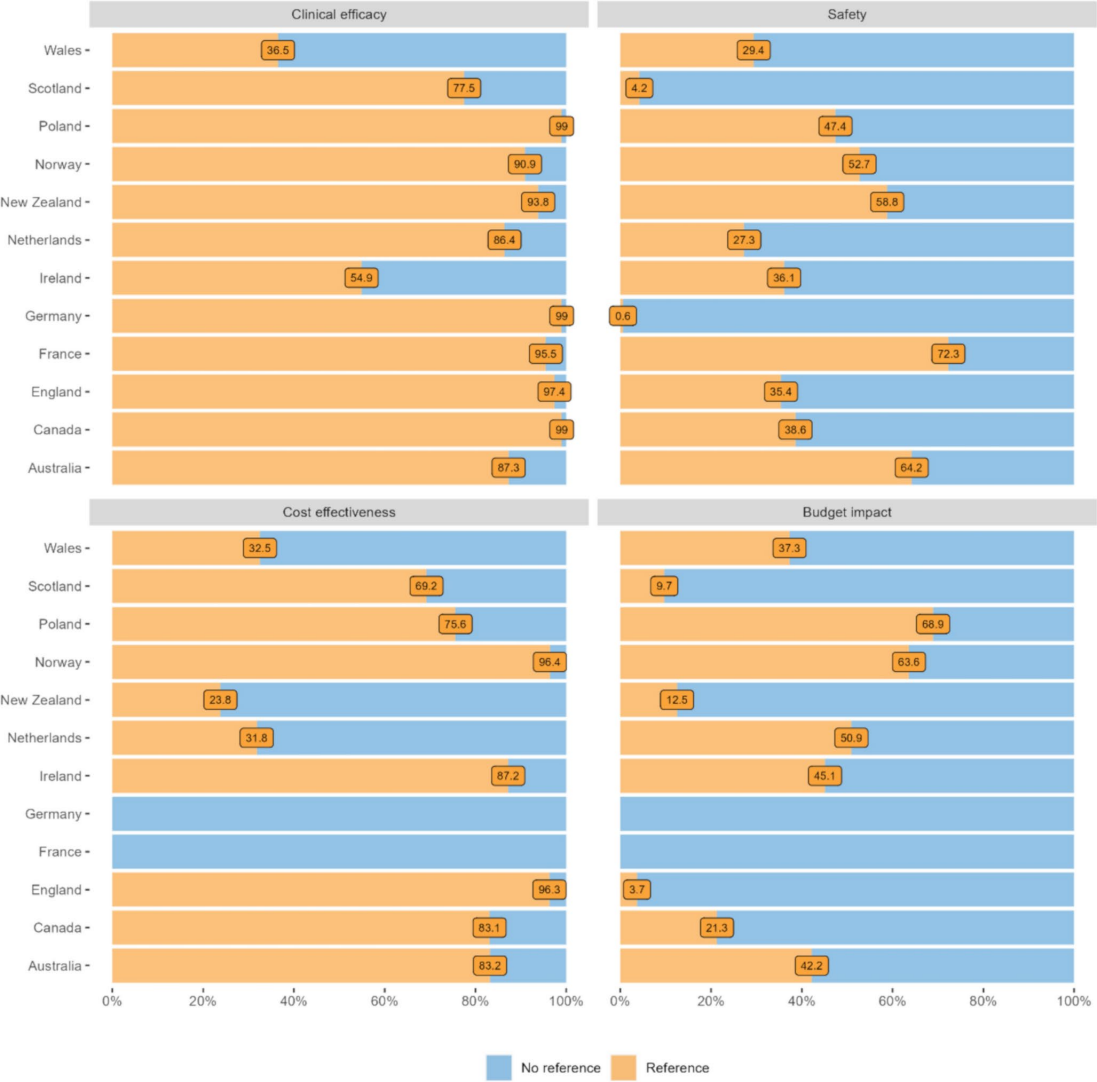
Percentage of quotations (references) by agency per country

**Fig. 3:**
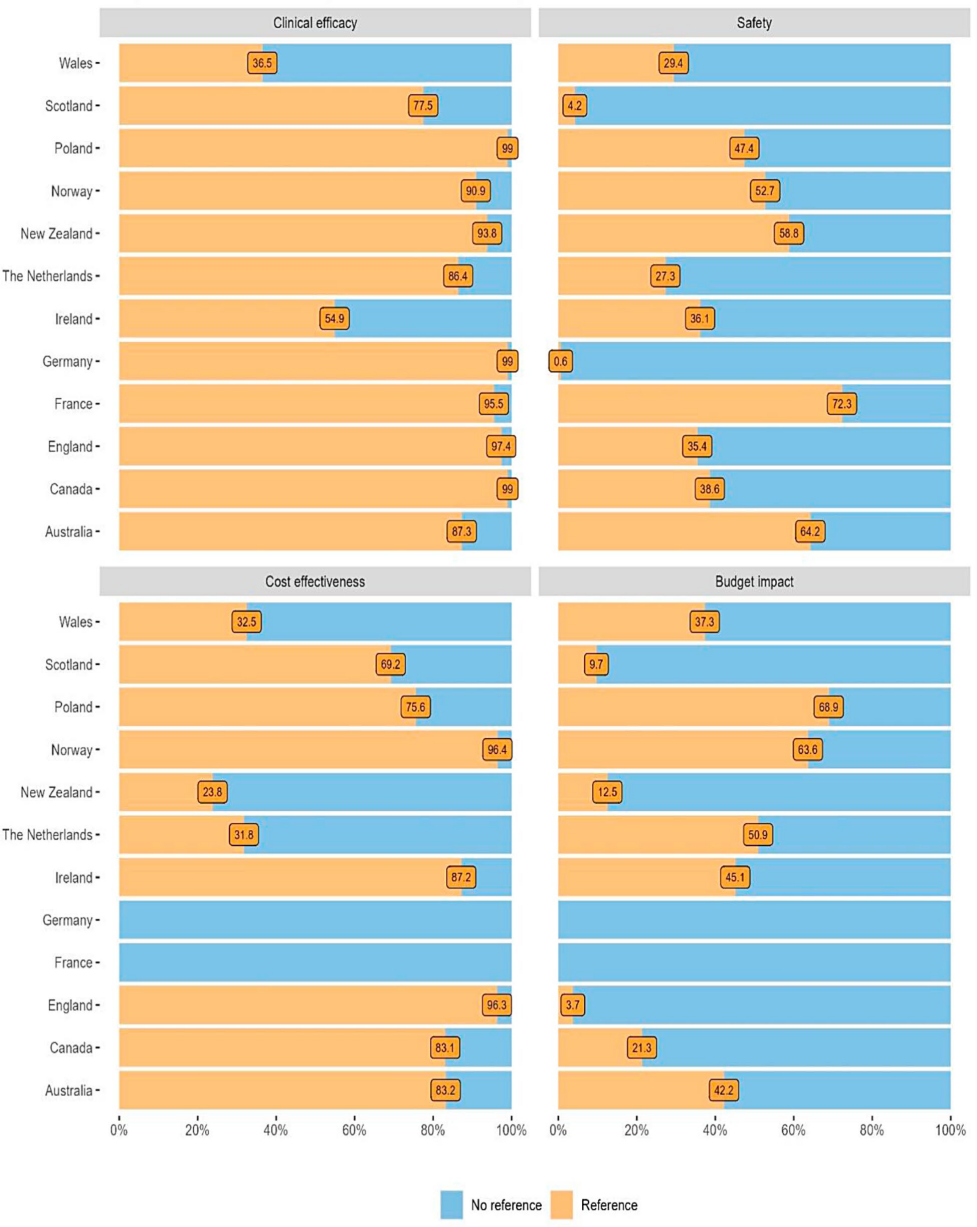
Percentage of quotations (references) by agency per country

The original article has been updated.

